# A rare case of acrodysostosis type 2 with PDE4D mutation in a young female: a case report

**DOI:** 10.1093/omcr/omae169

**Published:** 2025-01-18

**Authors:** Muhammad Sheraz Hameed, Maimoona Maheen, Sauban Mansoor Sadiq, Umer Farooq, Abdur Rehman, Arham Ihtesham, Imran Khan, Shahzaib Maqbool, Javed Iqbal

**Affiliations:** Department of Medicine, Rawalpindi Medical University, Tipu Road, Chamanzar Colony, Rawalpindi 46000, Punjab, Pakistan; Department of Medicine, Rawalpindi Medical University, Tipu Road, Chamanzar Colony, Rawalpindi 46000, Punjab, Pakistan; Department of Medicine, Shaheed Zulfiqar Ali Bhutto Medical University, Sector G-8/3, Ravi Road, Islamabad 44000, Pakistan; Department of Medicine, Rawalpindi Medical University, Tipu Road, Chamanzar Colony, Rawalpindi 46000, Punjab, Pakistan; Department of Medicine, Rawalpindi Medical University, Tipu Road, Chamanzar Colony, Rawalpindi 46000, Punjab, Pakistan; Department of Medicine, Rawalpindi Medical University, Tipu Road, Chamanzar Colony, Rawalpindi 46000, Punjab, Pakistan; Department of Medicine, PGY2 NYCH+H/Woodhull Brooklyn Network, 760 Broadway, Brooklyn, NY 11206, United States; Department of Medicine, Rawalpindi Medical University, Tipu Road, Chamanzar Colony, Rawalpindi 46000, Punjab, Pakistan; Nursing Department Communicable Diseases Center, Hammad Medical Corporation, Doha 3050, Qatar

**Keywords:** acrodysostosis, brachydactyly, nasal hypoplasia, pseudohypoparathyroidism

## Abstract

Acrodysostosis (ADO) is a rare form of peripheral dysostosis characterized by skeletal malformations, growth delays, short stature, and distinctive facial features caused by in part by underdeveloped (hypoplasia) of facial bones. Skeletal dysplasia is specific and includes disproportional short stature with short extremities and brachydactyly, multiple cone-shaped epiphyses, scoliosis or kyphosis with spinal stenosis, and advanced bone maturation. Herein, we are highlighting a case that presented with clinical features such as brachydactyly, delayed milestone, growth delay, muscle weakness and nasal hypoplasia. Patient genetic testing was in line with the diagnosis of acrodysostosis. The clinic-radiological correlation was also suggestive of the rare diagnosis of ADO.

## Introduction

Acrodysostosis (ADO) is a rare form of peripheral dysostosis that was initially described by Maroteaux and Malamut in 1968 [1]. It is characterised as a rare heterogeneous group of skeletal dysplasia that share characteristic features, including severe brachydactyly, facial dysostosis and nasal hypoplasia [2, 3]. Two types of ADO are known and has been separated by distinct references on OMIM database. ADO type 1 with or without hormone resistance (ACRDYS1, MIM 101800) is caused by pathogenic variants in chromosome 17 (locus 17q24.2) of the PRKAR1A gene. ADO type 2 with or without hormone resistance (ACRDYS2, MIM 614613) is associated with the PDE4D gene, which is located in the 5q11.2-q12.1 chromosomal region [4]. Characteristic clinical features include skeletal abnormalities, resistance to multiple hormones, and neurological involvement. Skeletal dysplasia is specific and includes disproportional short stature with short extremities and brachydactyly, multiple cone-shaped epiphyses, scoliosis or kyphosis with spinal stenosis, and advanced bone maturation. We report on a patient, who has clinical features of ADO type 2 described on the basis of clinical, radiological and hormonal parameters, though the genetic testing was also performed for the patients still the clinical parameters were good enough to correlate with the findings of ADO type 2.

## Case report

The patient, 17 years old female with no existing comorbids, presented in the outpatient department with a complaint of bilateral lower limb pain from 1 month that was progressive in nature involving both lower limbs, worsening since the last 15 days. Pain was also associated with bilateral lower limb swelling and tenderness in both legs followed by limitation of movements for one week. There was no history of fever or any other significant condition. The patient was vitally stable on her first presentation (Blood Pressure = 120/70 mmHg, pulse rate = 72b/min, temperature = 98°F, respiratory rate = 16/min, SPO2 = 98% at room air). On detailed history she had delayed mile stones; neck holding at 6 months, sitting at 9 months, speaking at 3 years, and walking at 5 years. She also had history of frequent fall with difficulty in combing hairs, changing her clothes, climbing stairs since age of 5 years, and stand holding on 5 years. Her sitting height was 28.7 inches, arm circumference is 25.2 inches with mini mental state score (MMSS) of 29/30. Menarche was appropriately started at 13 years of age, however the menstural cycle was reported to be irregular occasionally with mostly regular cycles.

**Figure 1:**
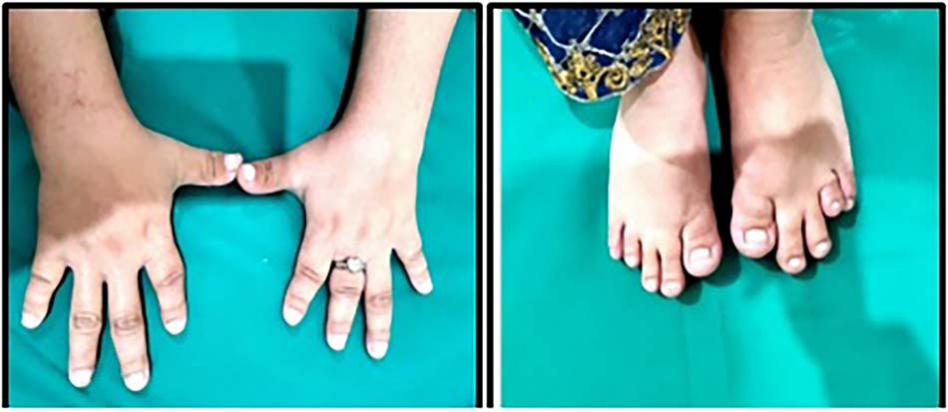
Brachydactyly of hands and feet.

On thorough musculoskeletal examination there was marked weakness, swelling, tenderness of both lower limbs. There was marked limitation of movement, and the fingers of hands and feet were also shorter than that of usual size (i.e. brachydactyly) and was congenital in nature as shown in Fig. 1. Overall, patient was also had short stature and bridging of the nose was also prominent.

**Figure 2:**
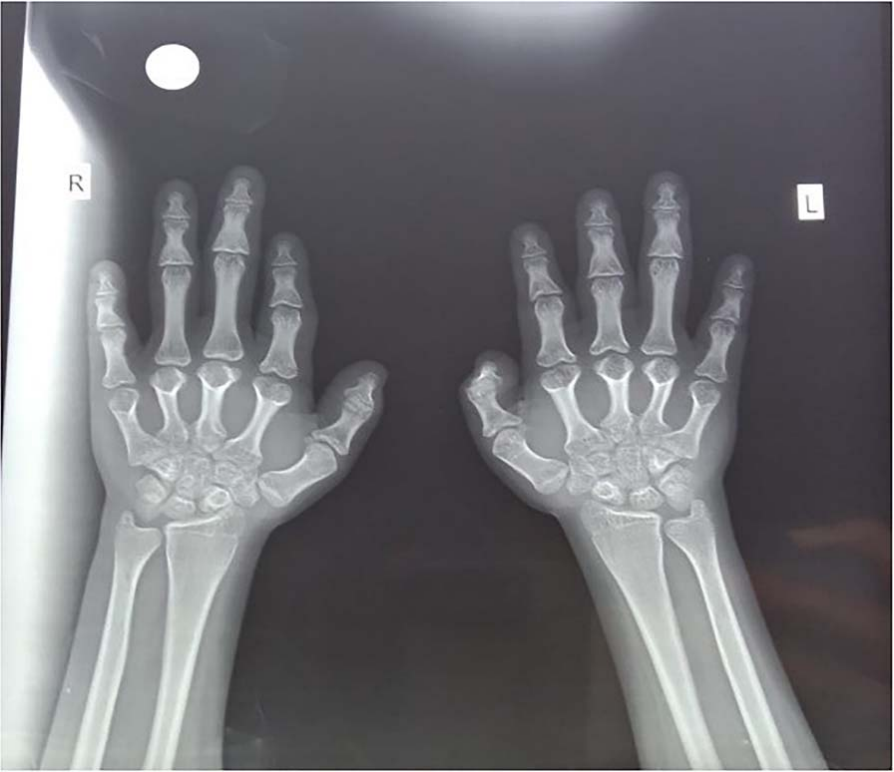
Showing reduced height of metacarpals and bone density.

The initial workup was performed and laboratory investigations were showing the Hb level of 9.9 mg/dl, white cell count (TLC) of 8.4, platelet counts of 509 and erythrocyte sedimentation (ESR) of 80. Similarly, the enzymatic profile including CPK, LDH, and aldolase was also performed showing the markedly increased CPK value of 592, however; LDH and aldolase were with in normal reference range. Serum PTH was 130 pg/ml (n = 15–65 pg/ml), 25-OH vitamin D was 65.2 nmol/l (n = 75–100 nmol/l), serum calcium was 2.35 mmol/l (n = 2.10–2.55 mmol/l), serum phosphorus 0.79 mmol/l (n = 0.74–1.52 mmol/l), serum glucose was 6.1 mmol/l (n = 4.2–6.1 mmol/l). X-rays of both hands and feet was also performed. X-ray of hands showed a reduced height of metacarpals with mild periarticular osteopenia and slight decreased bone density as shown in Fig. 2**.**

**Figure 3:**
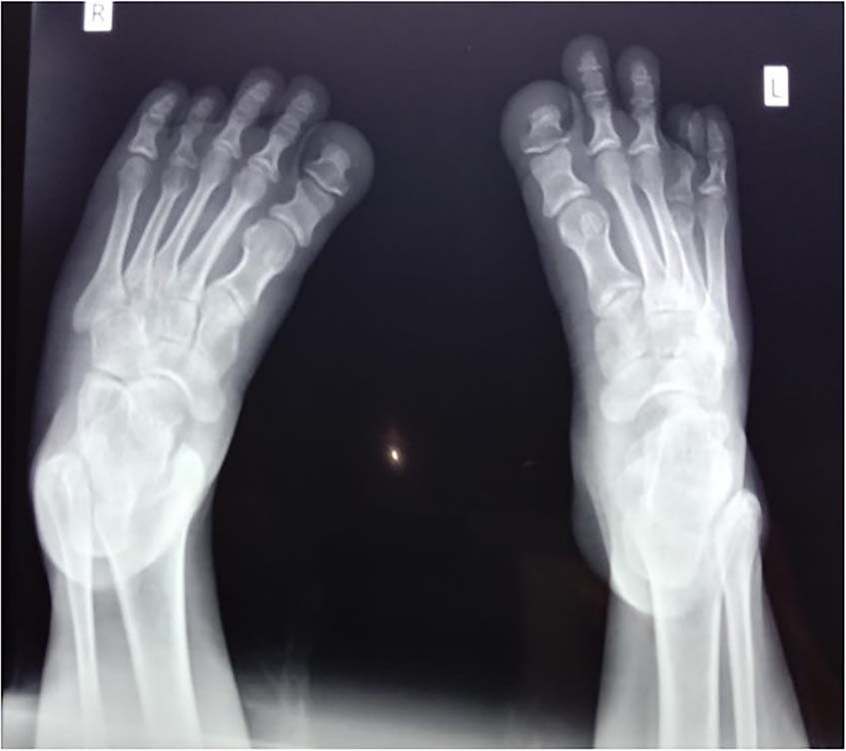
Showing reduced height of first and fourth metatarsals and bone density.

Similarly, the x-ray of feet was also performed that showed the reduced metatarsal height of first and fourth metatarsals with mild osteopenia and decreased bone density as shown in Fig. 3; however, these findings were also suggestive of pseudohypoparathyroidism as the differential diagnosis. Rest of the bone was normal with no lytic lesion, and reduced joint spaces and soft tissues swellings.

To get more insight of the case the muscle biopsy was performed that was consisted of multiple light brown pieces of tissues measuring 1.2 × 0.3 cm. The biopsy report was showing small muscle fibres with centrally located nuclei and some ring fibres were also seen. The overall impression of the muscle biopsy was suggestive of myopathy. All of the above mentioned clinical, radiological and histological findings were suggesting the rare case of ADO, though genetic testing was also done that showed PDE4D mutations. In the same way, the clinical and radiological profile of the patient was also showing the diagnosis of Acrodysostosis type 2. The patient was referred to endocrinologist for further management and intervention, however, the genetic counselling and follow-up visits were advised for multidisciplinary management.

## Discussion

ADO is rare genetic disorder characterized by skeletal malformations, growth delays, short stature, and distinctive facial features caused by in part by underdeveloped (hypoplasia) of facial bones [5]. The characteristic symptom of this disease is abnormally small hand and feet and short fingers and toes of hands and feet [6]. The patient we report falls much in line with these findings. The fact that these patients suffer from delayed milestones corroborates with our report as well. Our patient portrayed features like nasal hypoplasia, and nasal bridging that is characteristic of ADO [7]. The patient’s history also indicated delayed milestone achievement, frequent falls, and difficulty with activities such as combing her hair, changing clothes, and climbing stairs since the age of 5. She was able to stand while holding on at 5 years old. These findings are consistent with features of ADO, which presents not only with skeletal malformations but also with psychomotor and intellectual disabilities. [8].

ADO shares some clinical features with other disorders such as Albright hereditary osteodystrophy (AHO) and various skeletal dysplasias, which can complicate diagnosis. AHO, like ADO, presents with brachydactyly, short stature, and often intellectual disabilities. However, AHO is typically associated with hormonal resistance, particularly parathyroid hormone (PTH) resistance, leading to hypercalcemia and hyperphosphatemia, which were not evident in our patient [9]. Skeletal dysplasias can also manifest with short stature and limb abnormalities but often have distinct radiographic and genetic markers. In our case, the combination of delayed milestones, brachydactyly, short stature, and the characteristic facial features, along with the normal parathyroid function and specific radiographic findings, strongly suggest a diagnosis of ADO. This is further supported by the muscle biopsy indicating myopathy, a feature sometimes seen in ADO but not typically associated with AHO. While genetic testing is considered the gold standard for diagnosing ADO, the genetic analysis was in line with mutation of PDE4D gene. However, the clinical and radiological correlation was also sufficient to support the clinical diagnosis of ADO.

The prognosis for individuals with ADO can vary depending on the severity of skeletal malformations and associated complications. Long-term outlook often includes challenges related to physical development, intellectual disabilities, and potential hormonal imbalances. Regular follow-up is crucial to monitor growth, development, and any emerging health issues. Multidisciplinary care involving endocrinologists, orthopedic specialists, physical therapists, and developmental pediatricians is essential to manage the diverse aspects of the condition effectively and to improve the overall quality of life for the patient. A case report by Kartalias et.al emphasises a similar multidisciplinary approach for treatment of patients of skeletal dysplasias [10].

## Conclusion

In this report we presented a case of ADO in 17 years old girls who presented in the department of medicine with complaints of muscle pain, weakness, delayed milestones, delayed growth, reduced intellectual capabilities, brachydactyly, and nasal bone hypoplasia. All clinico-radiological correlations were attained. The diagnosis of ADO type 2 was made on genetic basis which showed mutation of PDE4D gene.
